# Curriculum Innovation: Virtual Reality for Neuroanatomy Education Among Neurology Residents

**DOI:** 10.1212/NE9.0000000000200359

**Published:** 2026-09-01

**Authors:** Ayush Gupta, Derek Bass, Christopher R. Barton, Wanyu Zhang, Maiying Kong, Daniela Terson de Paleville

**Affiliations:** 1 Division of Child Neurology, Department of Pediatrics, University of Arkansas Medical Sciences, Little Rock, AR;; 2 School of Medicine, University of Louisville, KY;; 3 Department of Pediatrics, Division of Child Neurology, University of Louisville, KY;; 4 Division of Gastroenterology Hepatology Nutrition, School of Medicine, University of Louisville, KY;; 5 Research Design, Compliance and Data Management Core, Louisville Clinical and Translational Research Center, University of Louisville, KY;; 6 Biostatistics Core, Brown Cancer Center, University of Louisville, KY;; 7 Department of Bioinformatics and Biostatistics, School of Public Health and Information Sciences, University of Louisville, KY; and; 8 Physiology Department, University of Louisville, KY.

## Abstract

**Introduction and Problem Statement:**

Advanced neuroanatomy knowledge is essential for clinical reasoning in neurology, yet residency training offers limited time to reinforce concepts. This contributes to persistent gaps in topics frequently tested on the Residency In-service Training Examination (RITE). This study evaluated the feasibility and educational impact of a virtual reality (VR) neuroanatomy curriculum intended to enable residents to (1) identify and spatially integrate arterial, venous, and ventricular structures; (2) improve performance on assessments targeting commonly missed RITE concepts; and (3) assess usability, engagement, spatial presence, and perceived educational value.

**Objectives:**

(1) To identify and label key neuroanatomical structures, (2) to describe spatial relationships relevant to clinical localization, (3) to apply neuroanatomy to clinical scenarios modeled after RITE items, and (4) to evaluate usability, engagement, and spatial presence of VR.

**Methods and Curriculum Description:**

A single-group, pre-post mixed-methods study among neurology residents at a single academic medical center. Participants completed a faculty-guided 40-minute VR session using Organon VR Anatomy on Meta Quest 3 headsets, focusing on cerebrovascular arterial supply, venous sinuses, ventricular anatomy, and key spatial relationships relevant to clinical localization. Knowledge was assessed using quizzes at baseline, postintervention, and at 2-week follow-up. Learner perceptions were measured using surveys, and data were analyzed using repeated-measures ANOVA and linear mixed-effects models, with mean differences and 95% confidence intervals reported.

**Results and Assessment Data:**

Thirty residents (mean age 30.5 years, SD 4.6; 50% female) completed all assessments. Mean scores increased from 8.57 (SD 2.75) at baseline to 11.37 (SD 2.27) immediately postintervention and were sustained at 11.20 (SD 3.07) at 2 weeks. The mean increase was 2.80 points (95% CI 1.45–4.15), representing an approximate 33% improvement, with sustained gains at follow-up (2.63 points; 95% CI 1.28–3.99). Participants reported high usability, engagement, and spatial presence.

**Discussion and Lessons Learned:**

This study demonstrates the feasibility of an instructor-guided VR neuroanatomy curriculum and provides preliminary evidence of improved knowledge and short-term retention. Findings should be interpreted in the context of a single-group design and nonidentical assessments, which limit causal inference. VR seems to be a promising adjunct to traditional instruction, particularly when combined with guided teaching. Future controlled studies are needed to evaluate comparative effectiveness and long-term clinical impact.

## Introduction and Problem Statement

Neuroanatomy education for neurology trainees is essential to their learning and clinical practice. Anatomical proficiency is a key component in the Residency In-service Training Examination (RITE).^1^ Knowledge of neuroanatomy is important to further understanding of neuroimaging and treating patients with epilepsy, stroke, and other neurovascular disorders. Understanding spatial relationships among brain structures helps neurologists make key decisions about diagnosis, pathology, and treatment, thereby significantly impacting clinical care. For example, localization in epilepsy can help determine targeted surgical treatments, stroke localization can assist in determining the appropriate next step, and correlation of neuroimaging with pathology can help generate differential diagnoses, to name a few.

Virtual reality (VR) is an evolving field with emerging data on its utility in medical education.^2,3^ VR-based anatomical education has been described as beneficial for surgical anatomy training in medical education^4,5^ A recent study suggested that VR has been increasingly used in medical student education, surgical training, and cardiovascular training.^6^ Similarly, the available literature on advanced VR curricula in neurology is limited to neurosurgery trainees and focuses on skill acquisition in that field.^7-9^ Attempts to improve RITE scores among neurology residents have been reported, including a recent paper on academic half day published recently.^10^ Participation in a 4-week multimodal bootcamp for neurology residents was not associated with improved scores.^11^ However, details of the curriculum are not available. Recent research highlighted ongoing efforts to improve structured neurology education and in-training examination performance. Furthermore, the effectiveness of using emerging technologies, such as VR, for neurology residents' education has not been studied.

A main caveat of the existing medical literature on the topic is its lack of detailed curriculum design and the implementation of pedagogical strategies.^12,13^ The existing literature on validated tools for the usability of VR technology in medicine is also limited.^14,15^ This study was conceived to address these gaps in education research. We developed and implemented a VR lesson to assess Neurology residents' knowledge of neuroanatomy concepts commonly missed on the RITE, using a pretest and posttest experimental design. We also studied whether a VR lesson leads to significant enjoyment and interaction without cyber fatigue, as well as the perceptions of neurology residents toward VR technology.

In this study, the VR intervention incorporated principles of retrieval practice, spaced learning, formative feedback, guided instruction, and cognitive load theory to promote active knowledge construction and long-term retention of complex neuroanatomical concepts. These instructional strategies were integrated into the experience through instructor-guided VR exploration, repeated application of anatomical concepts, and immediate feedback.

## Objectives

The curriculum was designed with 4 measurable learning objectives that guided both instructional design and learner assessment. The primary objective of this curriculum was to develop and implement an instructor-guided VR neuroanatomy curriculum for neurology and child neurology residents that reinforces high-yield neuroanatomical concepts commonly assessed on the RITE. A secondary objective was to evaluate the curriculum's educational effectiveness and learner experience. Specifically, the curriculum was designed to enable learners to

1. Identify and label key arterial, venous, and ventricular neuroanatomical structures.
2. Describe 3-dimensional spatial relationships relevant to neurologic localization.
3. Apply neuroanatomical knowledge to clinically relevant scenarios modeled after RITE-style questions.
4. Evaluate the usability, engagement, and spatial presence of the VR learning experience.

## Methods and Curriculum Description

### Participants

Thirty (n = 30) Neurology and Pediatric Neurology residents participated in this study. Exclusion criteria included inability to wear VR headsets and lack of consent. Residents from all training were encouraged to participate.

### Standard Protocol Approvals, Registrations, and Patient Consents

All procedures and protocols used in this study were reviewed and approved by the University of Louisville's Institutional Review Board (IRB #24.0474), in adherence with the institution's bioethical standards and guidelines. Participation was voluntary, and informed consent was implied through completion of the study surveys.

Although participation was voluntary, an information sheet was provided to the participants on enrolment, highlighting the benefits and risks of participating in the study. Consent was obtained, with the risks and benefits of exposure to VR technology explained. A $100 gift card was provided to each participant on completion of their sessions. The study was conducted between July 2024 and June 2025 at the Physiology Active Learning Lab (PAL Lab), in the Health Science Campus at the University of Louisville, where participants completed the VR training individually.

### Equipment

The VR headsets used in this study were the Meta Quest 3 and Meta Platforms, Inc. (Menlo Park, CA) both of which are commercially available. Participants were guided through the VR modules, while the instructor observed the mirrored display and provided real-time feedback (i.e., VR cast). The VR Software used for this study was Organon (Nicosia, Cyprus). 3-D Organon is a leading VR medical education software provider focusing on human anatomy and medical training through immersive VR experiences. This software is commercially available.^16^ Participants engaged with the VR environment using a head-mounted display (Oculus Quest), while their visual field was simultaneously projected (“cast”) in real time onto a laptop screen visible to the instructor. This allowed the instructor to observe the learner's perspective within the VR environment and provide synchronous verbal guidance.

### VR Protocol Design and Learning Objectives

Knowledge gaps were identified through a combination of prior resident performance trends on standardized RITE, faculty teaching experience, and informal learner feedback. One of the authors in this article, who is program director for the child neurology residency program, noted resident learners felt the need for improvement in RITE performance, particularly in neuroanatomy topics, because this was consistently the lowest average subcategory for child neurology residents on the RITE in consecutive years. Residents consistently highlighted challenges in understanding t3-dimensional neurovascular relationships. These gaps informed the selection and prioritization of anatomical content included in the curriculum.

The VR lesson plan and protocol were developed and pilot-tested with a small group of residents (n = 5) to identify and address potential technical pitfalls and refine the protocol.^17^ The curriculum was delivered over a minimum of 2 instructor-guided sessions lasting approximately 40 minutes each. The refined VR protocol used in this study encompassed 3 major learning topics for this project: anatomy of the intracranial arteries, venous structures, including the sinuses, and CSF flow. These were selected because they were identified as the major learning hurdles for early residents in our institution. Neuroanatomy accounted for 10% of the in-training questions, and these areas covered a significant proportion of them.^18^

The curriculum was developed collaboratively by a senior neurology trainee and the neurology residency program director both of whom have formal training and certification in Health Professions Education. The senior neurology trainee has additionally completed the American Academy of Neurology Neurologists as Educators program. AG demonstrated consistently high performance on the RITE, with scores above the 90th percentile over consecutive years, and had completed structured review of case-based neuroanatomy resources. The neurology residency program director has been actively involved in teaching residents and medical students and runs the residency program's weekly RITE preparation rounds. The VR environment and module adaptation were developed by investigators. The team evaluated multiple VR platforms and adapted a preexisting neuroanatomy module to align with neurology-specific learning objectives by curating relevant structures and removing extraneous content. Extraneous structures, such as the external carotid artery, the skull, the orbits, and other anatomical structures, were removed on one side to improve clarity. A key set of 30 structures was identified. In the modules, the 3-dimensional structures were in color, and 3 separate modules were designed, each for the arterial, venous, and ventricular structures. Individual structures can be identified by clicking on them with the VR controllers. The software is designed with an attached medical atlas (i.e., written information) and a side tab with literature for reviewing the structures. However, it was not used because of insufficient details, as determined during the pilot study. The module design was also informed by a previous pilot study that assessed the technology's feasibility and guided the refinement of both content and delivery.

Before the participants' arrival, immersive boundaries were established to provide ample space for interaction with the animations and to prevent injuries. Before the lesson began, the headset was fitted to each participant's head. Participants were asked to try the headset, and it was adjusted to ensure comfort during the VR experience. The headset was removed after this initial fitting, and they were asked to complete the pretest and demographic survey. Sessions were conducted in one-on-one or small groups of 2. The instructor directed learners through anatomical exploration, prompting identification of structures, and providing immediate corrective feedback. The instructor was also able to see the labels and was able to enhance the experience of learners by providing feedback on rotating or moving the structures for better quality and 3-dimensional orientation (through VR cast). The instructor did not directly control the VR interface but facilitated navigation and learning through verbal cues and interactive questioning. The researcher solicited feedback from participants about any adverse effects experienced while wearing the headsets, such as dizziness or loss of balance. The sessions could be conducted either standing or sitting, depending on the participant's preference.

The curriculum focused on 3 core neuroanatomical domains: arterial, venous, and ventricular anatomy. Key arterial structures included the internal carotid artery and its branches: middle cerebral artery (M1–M3 segments), anterior cerebral artery (A1–A2 segments), posterior cerebral artery (P1–P3 segments), and the vertebrobasilar system, along with high-yield branches such as the ophthalmic artery, artery of Percheron, posterior inferior cerebellar artery, and artery of Heubner. Venous structures included major dural venous sinuses and connecting veins (veins of Labbé and Rosenthal, vein of Galen). Ventricular anatomy encompassed the lateral, third, and fourth ventricles and relevant structures, including the choroid plexuses, foramina, thalamus, hypothalamus, and brainstem.

Within the VR environment, learners engaged in active 3-dimensional exploration by identifying anatomical structures, manipulating and rotating them to understand spatial relationships, and tracing vascular pathways. Structures could be interactively selected to display labels, facilitating real-time identification and reinforcement. Individual branches could be dragged out of the field of focus and reattached if needed. Instruction was delivered in a structured, stepwise manner: learners first identified structures, then explored their 3-dimensional relationships with one another, and subsequently applied this knowledge to clinically relevant contexts. Following these guided steps, learners were encouraged to explore additional structures independently and ask clarifying questions to reinforce their understanding. Gamification strategies were incorporated, allowing learners to disassemble and reconstruct anatomical structures (e.g., reassembling vascular branching patterns), thereby promoting active engagement and retrieval-based learning.

Clinical correlations were integrated directly within the VR session across all modules. In the arterial module, learners applied anatomical knowledge to vascular localization, including differentiation of middle cerebral artery superior and inferior division infarcts and their associated cortical territories, reasoning for equivalency of ophthalmic artery/central retinal artery infarcts with strokes, artery of Percheron infarcts and the imaging findings, and lateral and medial medullary syndromes in relation to vertebrobasilar anatomy and affected branches. In the ventricular module, learners explored patterns of hydrocephalus based on the site of obstruction, including congenital aqueductal stenosis and post-subarachnoid hemorrhage or postinfectious etiologies; intraventricular hemorrhage location in neonates based on the location of the choroid plexus; and the relationship of the Papez circuit to the lateral ventricles and adjacent structures. In the venous module, clinical vignettes included cavernous sinus thrombosis and associated cranial nerve involvement, superior sagittal sinus thrombosis cause frontal lobe hemorrhage, vein of Labbe thrombosis with associated temporal lobe infarction pattern, and vein of Galen malformation, with emphasis on 3-dimensional anatomical and neuroimaging correlations.

A comprehensive list of all structures included in the curriculum is provided in the supplemental materials (eTables 1 and 2). Throughout the sessions, the instructor reinforced connections between individual anatomical structures and their clinical relevance through integrated, vignette-based discussions.

The residents were taught about the origins, territories, drainage, and the 3-dimensional relationships of these structures. Clinical vignettes were added wherever possible to enhance retrieval practice. A gamification strategy was also used in which residents were asked to disintegrate and reconnect neurologic structures. The researcher solicited feedback from participants about any adverse effects experienced while wearing the headsets, such as dizziness or loss of balance. The sessions could be conducted either standing or sitting, depending on the participant's preference. The residents were to attend at least 2 visits of at least 40 minutes to complete the program, to support spaced retrieval learning, an evidence-based approach to promote knowledge retention.^19^

### Pretest and Posttest

To ensure comparability across assessments, pretest, immediate posttest, and 2-week follow-up questions were developed using a consistent content blueprint, with items mapped to the same neuroanatomical domains (arterial, venous, and ventricular anatomy). The immediate posttest and 2-week follow-up assessments used identical question sets to evaluate short-term retention. Although questions differed between the pretest and the posttest to minimize recall bias, they were designed to assess similar concepts and cognitive tasks. Question sets were developed collaboratively by the 2 neurologists on the research team; these questions were informed by a previous pilot study that used a similar assessment structure.^17^

The pretest questionnaires, consisting of 15 questions (listed in eTable 1), were administered to the residents at 3 time points: pretest, immediate posttest, and 2 weeks later. The questionnaire contained information pertaining to their demographics (age, sex, Neurology or pediatric neurology, and year in training); learner profile (do you consider yourself a visual learner, do you have exposure to VR before, year in training, study preference: textbook, flash cards, reading papers, or watching videos). In addition, the survey included self-reported assessments of confidence in neuroanatomy knowledge using a Likert scale from 0 to 100. Questionnaires were available on Qualtrics, and keys were prepared in advance of VR training. The assessment was designed to evaluate foundational and applied levels of learning based on Bloom's taxonomy, focusing on recall (identification of structures), understanding (three-dimensional spatial relationships), and application (clinical correlation and localization of neuroanatomical function). Higher-order domains such as evaluation and creation were not formally assessed within this curriculum.^19^

The posttest was completed at the end of the VR lesson (Table 1) and included additional questions about their perception of using VR on a Likert scale from 0 to 100 (0 = very unfavorable, 100 = very favorable) and about the ease of using VR (0–100). A previously standardized tool for assessing spatial presence in VR was also identified (Table 2).^20^ Participants were also asked whether VR should be included in the resident education curriculum. The posttest questionnaire was designed with a similar theme, and its difficulty was also assessed as moderate. Pretest and posttest items were thematically aligned but not identical; therefore, results should be interpreted as indicative of learning trends rather than as a precise measure of knowledge gain. Confidential comments were also sought from residents through the posttest questionnaire. A repeat questionnaire using the same set of questions was administered to assess memory and retrieval at 2 weeks after VR exposure. Full details of the Qualtrics-administered questionnaire are available in eTable 2.

**Table 1:** Comparisons of Total Scores Across 3 Time Points: Pretraining (Pre), Immediately Posttraining (iPost), and 2-Week Posttraining (2wPost)

Group	n	Pre mean (SD)	iPost mean (SD)	2wPost mean (SD)	*p* Value	iPost vs pre mean (SD)	2wPost vs pre mean (SD)	2wPost vs iPost mean (SD)
All participants	30	8.57 (2.75)	11.37 (2.27)	11.20 (3.07)	<0.001	2.80 (3.34)*	2.63 (3.16)*	−0.17 (3.17)
Age group (y)								
27–30.5	15	9.20 (2.54)	11.93 (1.75)	11.60 (1.96)	0.001	2.73 (2.99)*	2.40 (2.97)*	−0.33 (2.44)
30.5–44	15	7.93 (2.89)	10.80 (2.62)	10.80 (3.91)	0.006	2.87 (3.76)*	2.87 (3.42)*	0.00 (3.85)
Sex								
Women	15	8.80 (2.48)	10.80 (2.65)	11.20 (2.88)	0.010	2.00 (3.14)*	2.40 (3.00)*	0.40 (1.55)
Men	15	8.33 (3.06)	11.93 (1.71)	11.20 (3.34)	0.002	3.60 (3.44)*	2.87 (3.40)*	−0.73 (4.22)
Specialization								
Neurology	12	8.50 (2.88)	12.17 (1.53)	11.50 (3.55)	0.004	3.67 (3.20)*	3.00 (3.28)*	−0.67 (4.19)
Child neurology	18	8.61 (2.75)	10.83 (2.55)	11.00 (2.79)	0.003	2.22 (3.39)*	2.39 (3.15)*	0.17 (2.36)
Year in training								
1	4	9.00 (3.83)	11.25 (1.50)	11.50 (1.29)	0.295	2.25 (4.19)	2.50 (3.11)	0.25 (1.71)
2	7	9.43 (2.57)	12.14 (2.12)	10.57 (2.07)	0.157	2.71 (2.93)	1.14 (3.53)	−1.57 (3.87)
3	10	7.70 (3.13)	10.80 (2.66)	10.60 (3.95)	0.033	3.10 (4.25)*	2.90 (3.35)*	−0.20 (3.79)
4	6	8.67 (2.16)	11.67 (2.73)	12.00 (3.58)	0.031	3.00 (2.97)	3.33 (3.39)	0.33 (1.97)
5	3	8.67 (2.31)	11.00 (1.73)	12.67 (3.21)	0.083	2.33 (2.08)	4.00 (1.00)*	1.67 (3.06)
Visual learner								
Mainly a visual learner	22	8.45 (2.81)	11.45 (2.28)	11.50 (3.10)	<0.001	3.00 (3.52)*	3.05 (3.30)*	0.05 (3.37)
Identifies as a great visual learner	3	7.67 (3.21)	12.33 (1.53)	9.67 (1.53)	0.170	4.67 (3.51)	2.00 (3.61)	−2.67 (3.06)
Not a visual learner	5	9.60 (2.51)	10.40 (2.61)	10.80 (3.77)	0.363	0.80 (1.30)	1.20 (2.17)	0.40 (1.82)

*p* values were calculated using repeated-measures ANOVA to test whether total scores across the 3 time points were significantly different. The change scores between iPost vs pretraining, 2wPost vs pretraining, and 2wPost vs iPost by mean and SD are reported in the past 3 columns, where “*” indicates that a change is significantly different from zero under the repeated-measures ANOVA framework.

**Table 2:** Spatial Presence in Interactive Environment Questionnaire

Component	Score	Descriptor
1. Spatial Presence (SP)	5 (Excellent)	Strong feeling of immersion; user perceives themselves as “inside” the environment with high spatial awareness
	4 (Good)	Mostly immersive; minor inconsistencies detract from the “presence” experience
	3 (Average)	Moderately immersive; some spatial disorientation or lack of engagement occurs
	2 (Poor)	Low immersion: the user often feels detached from the environment
	1 (Unsatisfactory)	No sense of presence; the environment feels artificial or poorly rendered
2. Affordance and Performance (AFF)	5 (Excellent)	All features are intuitive and well-designed, and they significantly enhance task performance
	4 (Good)	Features are mostly effective, with minor usability challenges
	3 (Average)	Moderately effective; some features hinder task performance
	2 (Poor)	Limited affordances: significant effort needed to complete tasks
	1 (Unsatisfactory)	Poor design and affordances; tasks are challenging or impossible to complete
3. Enjoyment and Engagement (ENJ)	5 (Excellent)	Highly engaging and enjoyable; users actively participate with enthusiasm
	4 (Good)	Mostly enjoyable; minor elements reduce engagement
	3 (Average)	Moderately enjoyable; users occasionally disengage or lose focus
	2 (Poor)	Limited enjoyment: users frequently disengage
	1 (Unsatisfactory)	No enjoyment: experience is tedious or frustrating
4. Realness (REAL)	5 (Excellent)	Environment and representations are highly realistic and detailed
	4 (Good)	Mostly realistic, with minor inaccuracies or simplifications
	3 (Average)	Moderately realistic; noticeable discrepancies in detail or design
	2 (Poor)	Low realism: environment lacks authenticity
	1 (Unsatisfactory)	Unrealistic; the environment is unrecognizable or poorly rendered
5. Attention Allocation and Behavior (ATT)	5 (Excellent)	The tool strongly supports focus and guides user behavior effectively throughout tasks
	4 (Good)	Mostly effective at maintaining attention, with occasional distractions
	3 (Average)	Moderately effective; users are often distracted or disengaged
	2 (Poor)	Ineffective at maintaining focus; frequent distractions
	1 (Unsatisfactory)	Fails to support attention; users are unable to complete tasks
6. Perceived Cybersickness (CYB)	5 (Excellent)	No cybersickness experienced; the tool is highly comfortable
	4 (Good)	Minimal symptoms, easily tolerated without impacting performance
	3 (Average)	Moderate symptoms; occasionally disrupts focus or performance
	2 (Poor)	Significant symptoms: frequently disrupts the experience
	1 (Unsatisfactory)	Severe cybersickness; unable to continue using the tool

This table summarizes the scoring rubric used to evaluate 6 key components of the virtual reality (VR) learning experience: Spatial Presence (SP), Affordance and Performance (AFF), Enjoyment and Engagement (ENJ), Realness (REAL), Attention Allocation and Behavior (ATT), and Perceived Cybersickness (CYB). Each domain is rated on a 5-point scale (1–5), where higher scores indicate more favorable user experiences. Descriptors for each score level define qualitative criteria ranging from Excellent (5), reflecting optimal performance or experience, to Unsatisfactory (1), indicating substantial limitations such as low immersion, poor usability, limited realism, difficulty maintaining attention, or significant cybersickness. This framework supports systematic evaluation of user interaction, comfort, engagement, and perceived realism within the VR training environment.

### Data Availability

**Figure 1:**
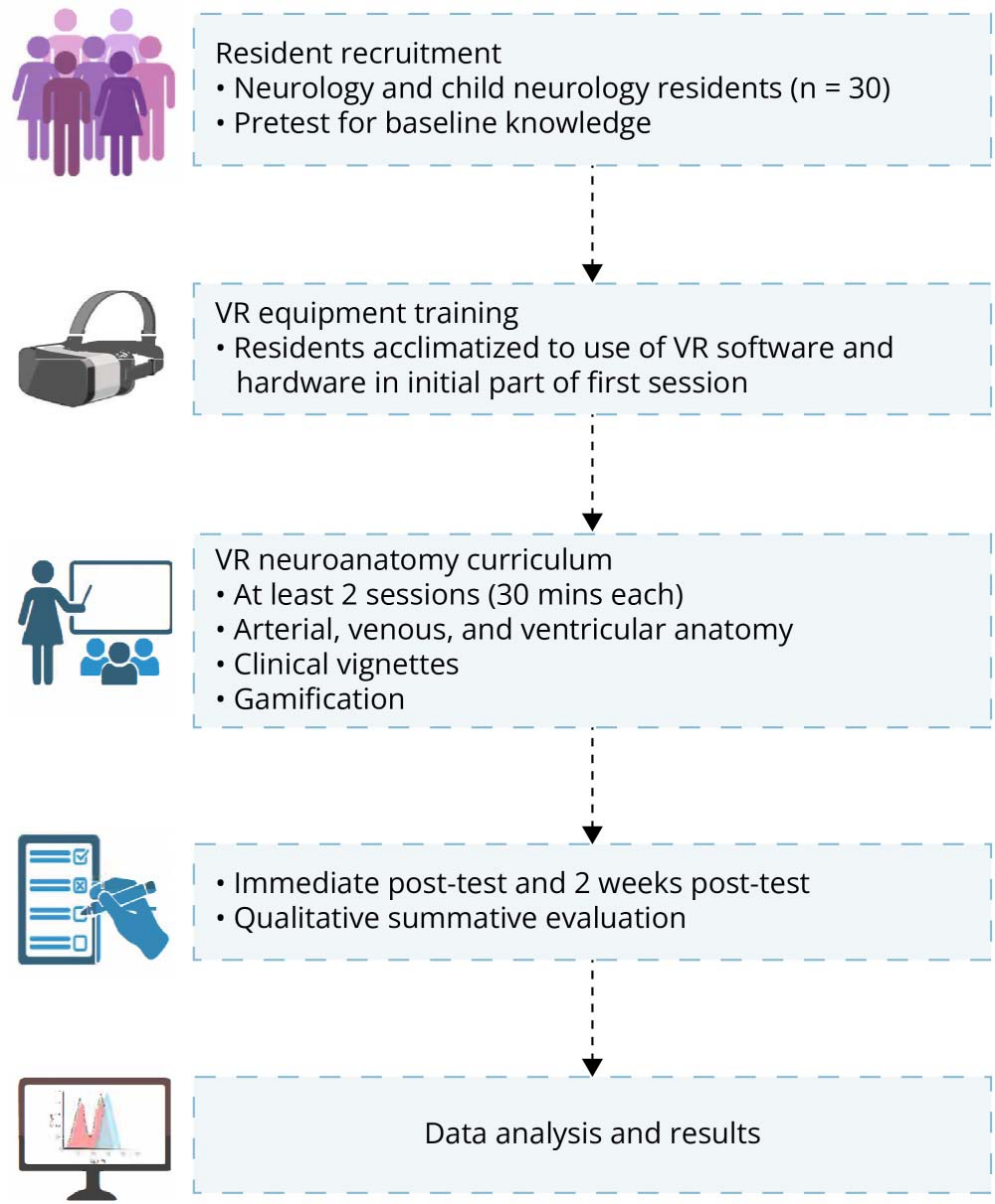
Sequential Phases of Virtual Reality (VR) Neuroanatomy Education Study for Neurology and Child Neurology Residents Participants first underwent recruitment and baseline knowledge testing, followed by an introductory session on VR equipment. The VR neuroanatomy curriculum included at least 2 30-minute sessions covering arterial, venous, and ventricular anatomy; clinical vignettes; and gamified learning modules. Residents then completed immediate and delayed posttesting as well as qualitative evaluations.

Anonymized data not provided in the article may be shared at the request of any qualified investigator for purposes of replicating procedures and results (Figure 1).

## Results and Assessment of Data

**Figure 2:**
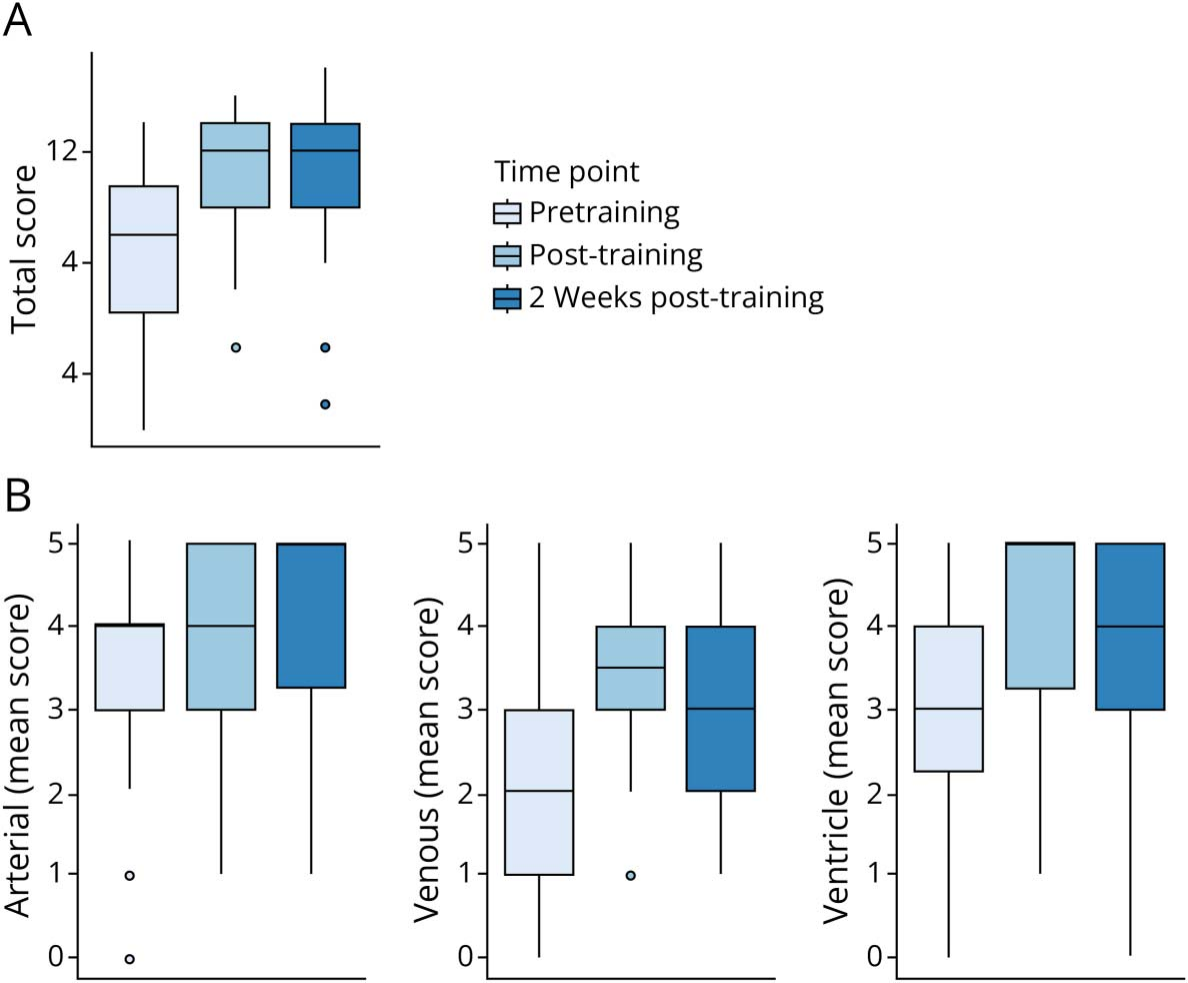
Assessment Scores Across Pretraining, Immediate Posttraining, and 2-Week Posttraining (A) Box plots illustrating changes in total assessment scores across pretraining, immediate posttraining, and 2-week posttraining time points. (B) Box plots depicting arterial, venous, and ventricular subscores over the same intervals.

Achievement of the curriculum learning objectives was evaluated using knowledge assessments and learner perception surveys. Learning objective 1 (identification of neuroanatomical structures) was demonstrated by significant improvements in arterial, venous, and ventricular assessment scores following the VR curriculum (Figure 2B). Learning objective 2 (understanding three-dimensional spatial relationships) was reflected by improvements across these anatomical domains and sustained performance at the 2-week follow-up. Learning objective 3 (application of neuroanatomical knowledge to clinical scenarios) was demonstrated by significant improvements in overall assessment scores, which increased from 8.57 ± 2.75 at baseline to 11.37 ± 2.27 immediately after training and remained elevated at 11.20 ± 3.07 two weeks later (*p* < 0.001). Learning objective 4 (evaluation of usability, engagement, and spatial presence) was supported by high learner ratings on the postintervention usability and spatial presence questionnaires, and positive qualitative feedback.

### Descriptive Statistics

Thirty residents (n = 30, 12 neurology, 18 child neurology) completed the VR neuroanatomy curriculum and all assessment time points. The mean age of participants was 30.5 years (range 27–44, SD 4.57), and the sex distribution was equal (15 women, 15 men). Nineteen of the participants were advanced learners, identified as being in year 3 or higher of residency training. Learning preference was only visual for 22 participants (n = 22), while 3 participants considered themselves as mainly visual learners, and 5 participants did not consider themselves as visual learners.

### Assessment of the Learning Objectives

Assessment of learning objectives was aligned with the curriculum goals. Objective 1 (identify and label neuroanatomical structures) was assessed through quiz items requiring identification of arterial, venous, and ventricular anatomy. Objective 2 (describe spatial relationships) was assessed through questions evaluating three-dimensional anatomical orientation and localization. Objective 3 (apply neuroanatomy to clinical scenarios) was assessed through case-based questions modeled after RITE items that required anatomical-clinical correlation. Objective 4 (evaluate usability, engagement, and spatial presence) was assessed using postsession learner perception surveys, including measures of usability, immersion, engagement, and spatial presence.

Content of the curriculum and assessments includes

1. Arterial vasculature of the brain, including territories of major blood vessels
2. Venous sinuses and venous drainage of the cerebral cortex and brain
3. Anatomy of the ventricles and CSF flow

### Quantitative Analysis

Figure 2 shows box plots of the total score and the Arterial, Venous, and Ventricular subscores at each of the 3 time points. Boxplots (Figure 2) show a marked rightward shift in total and subdomain scores immediately after the VR intervention, with scores remaining elevated at 2 weeks, indicating knowledge retention. The total scores across time points for the different subgroups are summarized in Table 1.

Repeated-measures ANOVA (rANOVA) revealed a significant effect of time on total neuroanatomy scores (*p* < 0.001).^21^ The mean score increased from 8.57 (SD 2.75) pretraining to 11.37 (SD 2.27) immediately posttraining and was maintained at 11.20 (SD 3.07) 2 weeks posttraining. This improvement was statistically significant and consistent both immediately and at follow-up (see the first row in Table 1). Subgroup analyses showed consistent improvements across age, sex, and specialization (neurology vs child neurology).The score increment was slightly higher among neurology residents than among child neurology residents, but both groups showed a statistically significant increase. Largest gains in scores were seen in PGY3 and PGY4 (PGY3: *p* = 0.03; PGY4: *p* = 0.03), although scores increased across all years; early trainees did not have a statistically significant increase in scores; their baseline scores were higher. Significant improvements were observed among residents who self-identified as strong visual learners (*p* < 0.001). These subgroup findings are exploratory and should be interpreted cautiously, given small sample sizes.

To assess the effect of time, while adjusting for age (continuous), sex (men, women), specialization (Neurology, Child Neurology), year in training (continuous), and visual learner (scale from 1 to 5), we fitted linear mixed-effects models with a random intercept for each participant, separately for the total score and for each area score. Because the overall time effects were significant, we conducted additional multiple comparisons with the pretraining score as the reference. The *p*-values for multiple comparisons are adjusted by the Bonferroni method and presented in Table 3. Subdomain of questionnaire analysis showed for total score, immediate posttraining vs pretraining difference was 2.80 (95% CI 1.45–4.15, *p* < 0.001), while 2-week posttraining difference was 2.63 (95% CI 1.28–3.99, *p* < 0.001). Significant improvements were observed across the arterial, venous, and ventricular subdomains (Figure 2B), demonstrating achievement of the curriculum objectives related to neuroanatomical identification, spatial understanding, and clinical application. Although the effect sizes in arterial domains were not as robust when compared between pre-exposure and immediate postexposure (*p* = 0.066) or 2-week follow-up (*p* = 0.004), the *p*-value for the overall time effect was statistically significant (*p* = 0.007). Venous subscore (*p* < 0.001) and ventricle subscore (*p* < 0.001) were both significant.

**Table 3:** Longitudinal Changes in Neuroanatomy Performance

	*p* Value for the overall time effect	Multiple comparison			
		Contrast	Estimate	95% confidence interval	*p* Value
Total score	<0.0001	Immediate post-pre	2.80	1.45 to 4.15	<0.0001
		2-wk post-pre	2.63	1.28 to 3.99	0.0001
Arterial score	0.0071	Immediate post-pre	0.57	−0.03 to 1.16	0.0657
		2-wk post-pre	0.83	0.24 to 1.43	0.0043
Venous score	0.0005	Immediate post-pre	1.07	0.40 to 1.74	0.0011
		2-wk post-pre	1.03	0.36 to 1.70	0.0016
Ventricles score	0.0006	Immediate post-pre	1.17	0.50 to 1.83	0.0003
		2-wk post-pre	0.77	0.10 to 1.43	0.0201

Comparisons of immediate posttraining vs pretraining, and 2-week posttraining vs pretraining, using a linear mixed-effect model with a random intercept for each participant, adjusting for age, sex, specialization, year in training, and visual learner status.

**Figure 3:**
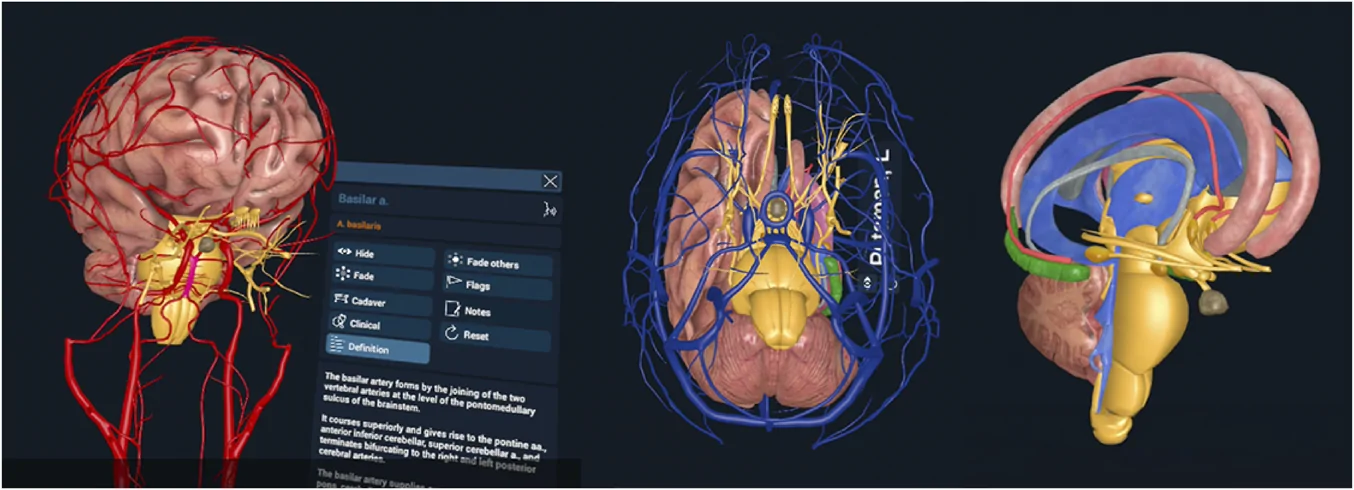
Virtual Reality Neuroanatomy Visualization Using the Organon Anatomy Platform Representative screenshots from the VR learning environment demonstrating interactive exploration of neurovascular and neuroanatomical structures. (Left) Posterolateral view emphasizing arterial circulation of the brain and brainstem, including the vertebrobasilar system, with the interactive annotation panel displayed. (Center) Inferior view highlighting predominantly venous vasculature and brainstem anatomy, enabling visualization of spatial relationships between major vessels and surrounding structures. (Right) Frontolateral view displaying deep brain structures, ventricular anatomy, and associated neural pathways, supporting three-dimensional understanding of internal neuroanatomical organization.

Pearson correlation coefficients between each subscore (arterial, venous, ventricles) and the total score demonstrated strong alignment at all time points (eTable 3). For example, the correlation between arterial and overall performance ranged from *r* = 0.74 to 0.81 across assessments, indicating that improvements in subdomains consistently contributed to overall gains (Figure 3).

### Qualitative Analysis

Thirty-three percent of participants (n = 10) provided confidential feedback through open-ended questions. Responses were organized by theme, as given in eTable 4. Notably, the feedback was positive, and learners seemed enthusiastic about VR as a part of the teaching curriculum. The feedback themes suggest an overwhelmingly positive reception, balanced by constructive suggestions for optimization. Overall, residents acknowledged the pedagogical value of immersive visualization. The enthusiastic endorsement for curricular integration was seen during the sessions and reflected in the comments. Residents explicitly noted that the ability to visualize structures from multiple perspectives and contextualize anatomical relationships significantly increased their motivation.

Important implementation challenges were also revealed, including physical discomfort associated with prolonged headset use, representing a practical barrier to sustained engagement and adoption. One participant experienced dizziness during initial use of the headset. This was recognized early, and the participant completed the session seated. One participant suggested a refinement to enhance intuitive interaction, a critical factor in immersive learning environments. Although VR was reported to be valuable for residents, the experience may have been designed at a cognitive and experiential level below that of neurology residents, who already possess foundational knowledge from clinical practice. This is specifically true for senior residents. The concern about the quality of a single-questionnaire item underscores an important reality: neuroanatomical variants are common and clinically relevant; thus, assessment tools must be dynamic enough to accommodate this neurobiologic reality. Collectively, these findings were interpreted as substantial promise of VR technology for neurology resident education. Successful implementation requires attention to ergonomic design, content calibration for different learner levels. The authors deemed these 2 key signals in the feedback.

## Discussion

This prospective pre-post mixed-methods study demonstrates the feasibility of a personalized, instructor-guided virtual reality (VR) curriculum for neuroanatomy education among neurology and child neurology residents and provides preliminary evidence of improved knowledge and favorable learner perceptions. The intervention was conceptualized as a technology-enhanced, instructor-mediated educational model. These findings suggest that instructor-guided VR may enhance short-term acquisition and retention of neuroanatomy knowledge. Observed improvements were educationally meaningful and sustained over 2 weeks.

In our study, qualitative feedback from residents was highly positive, with participants consistently emphasizing improved three-dimensional spatial visualization and enhanced understanding of complex intracranial vascular relationships. The SP-IE questionnaire supported high levels of engagement, spatial presence, and perceived educational value. Residents endorsed integration of VR into the curriculum while also identifying areas for refinement, such as interface usability and the need for greater depth of content for advanced learners. Our study highlights that, in addition to didactic and experiential learning through clinical practice, VR can serve as an adjunct to improve knowledge and clinical application of neuroanatomy. This is a highly relevant topic in the current context educational environment, as the costs of other tools, such as cadaveric teaching become dissection, are becoming prohibitively expensive and present ethical challenges.^22^ Although VR exposure for training has long been successfully used in simulations in other industries (aviation), for aircraft pilot training with good results.^23^ However, the use in medicine has only recently been recognized. Previous studies in medical students and surgical trainees have reported improvements in learning outcomes and user engagement, although heterogeneity in study designs limited any causal inference. Previous research on VR Neuroanatomy has included medical students, neurosurgeons in training, surgical residents, and social science students.^7-9,24,25^ The results from medical student studies were largely similar to ours, showing higher scores and retention than with traditional textbook training. A randomized controlled trial by Ekstrand et al. involving first-year and second-year medical students demonstrated that VR neuroanatomy use in medical education achieved adequate knowledge outcomes, high-quality engagement, and endorsement by learners. Further, immersive VR neuroanatomy training achieved knowledge outcomes equivalent to paper-based methods while significantly enhancing engagement, reducing “neurophobia” (81% of participants), and garnering near-universal endorsement for curricular integration (94%).^3^ The improvement in scores was about 20% in the VR exposure group in this study, which is similar to our cohort. Similar experiences have been reported in VR use in neurosurgical trainees; however, such research was more focused on procedural skills related to neuroanatomy rather than clinical knowledge application and localization-focused approach in our curriculum. Therefore, direct comparison of this study with our study is not possible. In other available literature, a recent metanalysis also highlighted heterogenous design and a largely nonrandomized body of literature in context of VR curriculum for medical education. This led to moderate effect size and lack of causal inference. A more recent meta-analysis suggested that the studies had different comparators and were mostly nonrandomized.^26^ The effect size was moderate, and causal inference could not be established because of heterogeneity. Our findings extend this literature to neurology trainees and support the use of VR as an adjunct to existing educational approaches.

In this study, we not only implemented a VR-based module but also implemented it with education-based principles, including formative assessment and feedback, retrieval practice, spaced learning, creation of conducive learning environments, and use of structured approach. These strategies align with the principles of cognitive load theory and deliberate practice, suggesting that the observed learning gains likely reflect the combined effect of immersive visualization and guided instruction. This approach contrasts with the predominant methodology in existing literature. Our pilot study had also suggest the need for an expert trainer to improve knowledge and skill acquisition.^16^ This approach is more consistent with collaborative VR models previously described.^7^ These facilitated sessions, as in our case, suggest that the educational value of VR may be maximized when combined with active teaching rather than used as a standalone self-study tool. Our study also highlights the need of integration of educational principles with technology usage. The duration and structure of our intervention also differed from those in prior literature. In our pilot study,^16^ we demonstrated that residents were familiar with VR and did not need a training session. Our strategy of completing 2 learning sessions differed from what was reported in the literature previously. Our strategy differed from that reported in the literature. For example, a randomized controlled trial of Ekstrand et al. provided medical students with only 12 minutes of self-paced VR exploration without active teaching or feedback.^3^ The combination of expert-led instruction, formative feedback, extended exposure, and clinical contextualization may partially explain why our study demonstrated sustained knowledge retention at 2-week postintervention.

The findings from this study open several important avenues for future investigation; one of the most pressing research needs is to evaluate long-term retention and transfer of learning to clinical practice. Studies are needed to determine whether knowledge persists throughout residency training and influence clinical decision-making in real-world patient encounters. Given that VR technology remains expensive, a cost-benefit analysis is required to compare this approach with other learning modalities, including didactic and lab instruction, as well as clinical teaching. Future studies should assess whether residents who complete VR neuroanatomy training demonstrate superior performance on neuroimaging interpretation, stroke localization accuracy, or epilepsy surgical candidacy evaluations when compared with traditionally trained peers. These outcomes are needed to study VR's impact on clinical competence beyond knowledge acquisition. Collaborative efforts involving national neurology organizations could significantly increase the effect of similar studies on educational curriculum design for advanced learners.

This study has some limitations that warrant consideration when interpreting the findings. First, the single-center design with a relatively small sample size limits generalizability to neurology residency programs. The absence of a control group receiving traditional neuroanatomy instruction represents a significant methodologic limitation inherent to our pre-post design. Although we demonstrated statistically significant improvements in test scores following VR exposure, the exact contribution of VR intervention cannot be definitively described. Confounding factors such as the Hawthorne effect, natural knowledge accumulation, and the effect of the utilization of learning theories should be accounted for in score gains.^27-29^ A randomized controlled trial with parallel groups, receiving VR-based vs traditional didactic neuroanatomy instruction, would provide stronger causal evidence.

An important limitation relates to the validity of assessment tools. Although preintervention and postintervention assessments were constructed using consistent content blueprint and designed to evaluate similar concepts, they did not consist of identical or formally validated parallel forms. The absence of formal psychometric analysis and variability in item structure may introduce uncertainty in interpreting the magnitude of observed score changes. As such, the findings should be interpreted as reflecting trends in knowledge acquisition rather than definitive measures of learning gains attributable solely to the intervention. Sessions were conducted by a single instructor, which could lead to confounding bias. Self-selection bias may have influenced our results because participation was voluntary and included an incentive of 100 dollars and may have induced a selection bias by attracting residents with higher technologic aptitude or intrinsic motivation and skewing the participant population. Future studies should incorporate multicenter designs with larger use validated parallel assessment instruments and include comparison groups receiving traditional or alternative instructional modalities. Randomized or controlled study designs will be essential to better isolate the independent effect of VR-based instruction and to evaluate its effect on long-term knowledge retention and clinical performance.

## Lessons Learned

Several practical “lessons learned” emerged from implementation of this curriculum that may inform educators considering the integration of VR into residency education. First, immersive technology alone is unlikely to maximize learning; instructor-guided facilitation, immediate formative feedback, and structured questioning seemed to be essential components of the educational experience. Second, aligning VR activities with evidence-based instructional strategies, including retrieval practice, spaced learning, and clinical contextualization, may enhance knowledge acquisition and retention beyond what visualization alone can provide. Third, tailoring content to the learner's level of training is important because senior residents expressed a need for greater complexity, while junior learners appeared to benefit most from foundational three-dimensional visualization. It is important that lessons using VR may require a dedicated, stable internet connection and a strong signal or access, particularly if multiple VR headsets are used simultaneously. Institutions often have firewalls or internet security systems that may affect the ability to use VR. Therefore, it is important for educators to familiarize themselves in advance with the technical capabilities available at the institution and access to technical support (e.g., University IT and educational technology). Finally, educators should consider practical implementation factors such as learner comfort, session duration, and faculty development when incorporating VR into graduate medical education.

In summary, our study provides evidence that VR-based neuroanatomy education could be an effective adjunctive tool for neurology residents, with gains in knowledge and engagement. The pre-post intervention curriculum showed excellent enjoyment and no significant cyber fatigue. These findings support the extension the VR education literature beyond foundational undergraduate learning to specialist-level training. We highlight VR's potential as a scalable, engaging, and pedagogically sound tool for advanced neuroanatomy education when appropriately tailored to learner needs and clinical context. Qualitative analysis revealed significant enthusiasm, but a need to improve module quality, provide greater detail, and include an experienced teacher to support the usability and comfort of VR technology. Our qualitative findings support the recommendation that VR should be viewed as an enhancement to, rather than a replacement for expert teaching, with technology serving as an important tool that amplifies the effectiveness of skilled instruction. Further studies are needed to compare different learning strategies and techniques in neurology education and determine optimal implementation strategies that balance educational effectiveness with the practical constraints of faculty time and availability.

## Glossary

RITE: Residency In-service Training Examination
VR: virtual reality
